# Association of Serum Alkaline Phosphatase with the TG/HDL Ratio and TyG Index in Korean Adults

**DOI:** 10.3390/biom11060882

**Published:** 2021-06-14

**Authors:** Da-Hye Son, Hyun-Su Ha, Yong-Jae Lee

**Affiliations:** 1 Department of Family Medicine, Yonsei University College of Medicine, Seoul 03722, Korea; sonda@yuhs.ac; 2 Department of Integrative Medicine, Yonsei University Graduate School, Seoul 06229, Korea; 3 Department of Medicine, Yonsei University Graduate School, Seoul 03722, Korea; hsha@yuhs.ac

**Keywords:** alkaline phosphatase, insulin resistance, triglyceride and glucose index, triglyceride to high-density lipoprotein cholesterol ratio

## Abstract

Alkaline phosphatase (ALP) has long been considered a marker of hepatobiliary and bone disorders, but recent studies have shown that increased ALP activity is correlated with various cardio-metabolic diseases. Thus, we investigated the association of serum ALP level with surrogate markers of insulin resistance such as triglyceride to high-density lipoprotein cholesterol ratio (TG/HDL-C ratio) and triglyceride and glucose (TyG) index in the general population. The study included 12,868 men and women aged 19 years and older. Participants were categorized into four groups based on serum ALP level (U/L) as follows: Q1: 55–190 U/L, Q2: 191–224 U/L, Q3: 225–265 U/L, and Q4: 266–923 U/L for men, Q1: 48–161 U/L, Q2: 162–198 U/L, Q3: 199–245 U/L, Q4: 246–790 U/L for women. The insulin resistance cut-off levels were defined corresponding to the 75th percentile of the TyG index and TG/HDL-C ratio in the current samples. Odds ratios (ORs) with 95% confidence intervals (CIs) of insulin resistance according to quartile of serum ALP level were calculated using weighted multivariate logistic regression analysis. Compared with Q1, the adjusted OR (95% CI) for insulin resistance of the Q4 serum ALP group was 1.517 (1.234–1.866) in men and 1.881 (1.399–2.528) in women using the TG/HDL-C ratio and 1.374 (1.093–1.728) in men and 2.047 (1.468–2.855) in women using the TyG index after adjusting for confounding variables. Serum ALP levels are independently and positively associated with surrogate markers of insulin resistance in Korean adults.

## 1. Introduction

Insulin, which is secreted from pancreatic beta cells, is the main hormone that regulates cellular metabolism in many tissues in human body. Insulin resistance is characterized by impaired glucose uptake and oxidation, a decrease in glycogen synthesis, and a loss of function to suppress lipolysis. The clinical importance of insulin resistance has emerged due to its association with cardio-metabolic diseases, such as type 2 diabetes, cardiovascular disease (CVD), hypertension, nonalcoholic fatty liver disease (NAFLD), and metabolic syndrome [1,2,3,4]. In particular, insulin resistance plays a key role in the development of metabolic syndrome. Although the gold standard measurement for assessing insulin resistance is hyperinsulinemic-euglycemic clamp, it is rarely used in a clinical setting due to high cost, low accessibility, and low reproducibility [5]. In this context, alternative indicators of insulin resistance have been documented in previous studies [6,7,8]. The triglyceride to high-density lipoprotein (HDL)–cholesterol ratio (TG/HDL-C ratio) and triglyceride and glucose (TyG) index are commonly used as alternative markers for assessing insulin resistance.

Alkaline phosphatases (ALPs) are a group of isozymes catalyzing the hydrolysis of organic phosphate esters at basic pH [9]. Human ALPs are classified into at least four isozymes according to specificity of the tissue to be expressed as follows: intestinal alkaline phosphatase, germ cell ALP, placental ALP, and liver/bone/kidney ALP, which is also called tissue-nonspecific ALP (TNSALP) [10]. Various causes can contribute to increase in serum ALP level, but TNSALP constituted about 90% of the total serum ALP activity [11]. Thus, ALP activity has long been used as a marker of hepatobiliary and bone disorders [12]. However, recent studies have demonstrated that increased ALP activity is related to various cardio-metabolic diseases [13,14,15,16,17]. Kim et al. showed a positive, independent association between metabolic syndrome and serum ALP even after adjusting for potential confounding factors [15]. The link between serum ALP and metabolic syndrome is unclear, but chronic low-grade inflammation and insulin resistance appear to contribute. In our previous study, we showed that serum ALP level was positively associated with a high level of inflammatory markers such as leukocyte count and C-reactive protein in elderly Koreans [18]. However, little is known about the association between serum ALP level and biomarkers of insulin resistance. Thus, we investigated the association of serum ALP level with surrogate markers of insulin resistance such as triglyceride to high-density lipoprotein cholesterol ratio (TG/HDL-C ratio) and triglyceride and glucose (TyG) index in the general population using data from the KNHANES dataset.

## 2. Materials and Methods

### 2.1. Study Population

**Figure 1:**
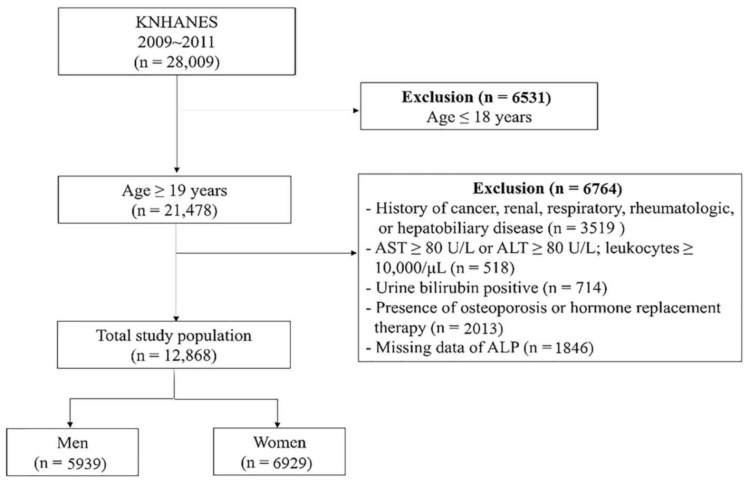
Flowchart of the study population selection.

This cross-sectional study analyzed data obtained from the Korean National Health and Nutrition Examination Survey (KNHANES) provided by the Korea Centers for Disease Control and Prevention (KCDC) from 2009 to 2011. KNHANES is a nationwide, representative, population-based survey that assesses the nutritional and health status of Koreans. Data sampling was performed using a stratified, multi-staged, probability sampling design based on sex, age, and geographical area via household registries. Of the 28,009 participants enrolled in the survey during the study period, we excluded those who met the following criteria: children and adolescents aged ≤18 years (*n* = 6531); presence of osteoporosis; history of cancer, renal, respiratory, rheumatologic, or hepatobiliary disease; AST ≥ 80 U/L or ALT ≥ 80 U/L; leukocytes ≥10,000/μL; positive urine bilirubin; missing data of ALP, or history of hormone replacement therapy. After excluding these individuals, 12,868 participants were included in the final analysis (Figure 1). The average age of this study population was 46.4 years, and the oldest individual was 80 years.

### 2.2. Data Collection

The 2009–2011 KNHANES contained health, nutritional, social, and demographic data obtained through a three-component survey method. Anthropometric measurements were measured by trained medical staff following a standardized procedure. Body weight and height were measured to the nearest 0.1 kg and 0.1 cm, respectively, in light indoor clothing without shoes. BMI was calculated as weight in kilograms divided by square of height in meters (kg/m^2^). SBP and DBP were measured using the patient’s right arm while the patient was seated and after 10 min of rest, using a standard mercury sphygmomanometer (Baumanometer, W.A. Baum Co Inc., Copiague, NY, USA). Self-reported cigarette smoking, alcohol consumption, and physical activity characteristics were collected from questionnaires. Current smoker was defined as a person who currently smokes and who has smoked more than 100 cigarettes during their lifetime. Ex-smoker was defined as a person who quit smoking but has smoked more than 100 cigarettes during their lifetime, and non-smoker was defined as a person who has never smoked. Questions about alcohol intake included weekly frequency. Regular alcohol consumption was defined as alcohol drinking ≥ twice per week. The resistance exercise group was defined as those who performed resistance exercise ≥ three times per week. Educational level was categorized as either elementary school or below, middle school or below, high school, and college or above. Household income was classified into four quartiles from lowest to highest. The Homeostatic Model Assessment for Insulin Resistance (HOMA-IR) value was calculated using the following formula: fasting plasma glucose (mg/dL) × fasting insulin (μIU/mL). Metabolic syndrome was defined as the presence of at least three of the following criteria according to the National Cholesterol Education Program Adult Treatment Panel III (NCEP-ATP III): high blood pressure (SBP ≥ 130 mmHg or DBP ≥ 85 mmHg); central obesity according to the Asian-Pacific criteria (waist circumference ≥90 cm for men and ≥80 cm for women); high fasting glucose (≥100 mg/dL); high TG (≥150 mg/dL); low HDL-cholesterol (<40 mg/dL for men or <50 mg/dL for women). Individuals who reported taking anti-hypertensive medication or anti-diabetes medications were considered to have elevated blood pressure or elevated fasting glucose. Obesity was defined as BMI ≥ 25.0 kg/m2 according to World Health Organization (WHO) cut-off levels for adult Asians [19]. All blood samples were obtained from the antecubital vein after a 12 h overnight fast. Fasting plasma glucose (reference range: 70–99 mg/dL), ALP (reference range: 40–160 U/L), ALT (reference range: ≤35 U/L), AST (reference range: ≤40 U/L), triglyceride (reference range: <150 mg/dL), and HDL cholesterol levels (reference range: >60 mg/dL) were assessed using a Hitachi 7600 automated chemistry analyzer (Hitachi Co., Tokyo, Japan) with enzymatic assays following the International Federation of Clinical Chemistry and Laboratory Medicine (IFCC) recommendation. Leukocyte count was assessed by an automated blood cell counter (XE-2100D; Sysmex, Kobe, Japan).

### 2.3. TyG Index

TyG index was defined as

*TyG = Ln [fasting triglyceride (mg/dL) × fasting plasma glucose (mg/dL)/2]*

### 2.4. Statistical Analysis

Serum ALP quartiles were categorized as follows: Q1: 55–190 U/L, Q2: 191–224 U/L, Q3: 225–265 U/L, and Q4: 266–923 U/L for men, Q1: 48–161 U/L, Q2: 162–198 U/L, Q3: 199–245 U/L, Q4: 246–790 U/L for women. Clinical characteristics of the study population according to serum ALP quartile were compared using one-way analysis of variance (ANOVA) or Kruskal–Wallis test for continuous variables according to normality of distribution and chi-square tests for categorical variables. Results are expressed as mean and standard deviation (SD) or number (percentage) for quantitative variables. The ORs (95% CIs) for high TG/HDL ratio and high TyG index were calculated using multiple logistic regression analysis after adjusting for confounding variables across ALP quartiles. To control for type I error, we made the *p*-value more stringent using bonferroni correction. Significant *p*-value for post hoc was determined at *p*-value < 0.0167. High TG/HDL ratio and TyG index were defined as those greater than 3.65 and 4.83, respectively, corresponding to the 75th percentile of the current samples. Proportion of insulin resistance according to serum ALP quartiles were compared using chi-square tests. All analyses were conducted using SPSS statistical software (version 25.0; SPSS Inc., Chicago, IL, USA). All statistical tests were two-tailed, and statistical significance was determined at *p*-value < 0.05.

### 2.5. Ethics Statement

The study protocol was reviewed and approved by the Institutional Review Board of the KCDC and Prevention (IRB No. 2009-01CON-03-2C, 2010-02CON-21-C, 2011-02CON-06-C). Written informed consent was obtained from all participants when the KNHANES was conducted, in accordance with the ethical principles of the Declaration of Helsinki.

## 3. Results

### 3.1. Clinical Characteristics of the Study Population

The clinical characteristics of the study population according to serum ALP quartile are presented in Table 1. SBP, DBP, insulin, HOMA-IR, triglycerides, AST, ALT, and white blood cell (WBC) count significantly increased from the lowest to highest ALP quartiles in both sexes, but mean age, BMI, fasting plasma glucose, HDL-C significantly increased according to ALP quartiles only in women. Proportions of individuals with metabolic syndrome significantly increased according to ALP quartiles in both sexes, but the proportions of hypertension, impaired fasting glucose, diabetes mellitus, and obesity significantly only increased in women. Additionally, high household income and high education level were significantly lowest in the highest ALP quartile.

### 3.2. Association between TG/HDL Ratio and ALP

**Figure 2:**
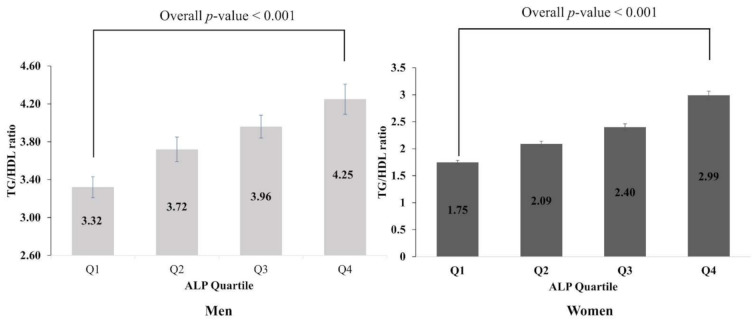
Trend analysis of TG/HDL ratio according to ALP quartile in both sexes. Values are assessed by ANOVA.

Trend analysis of TG/HDL ratio according to ALP quartile in both sexes is shown in Figure 2. The mean TG/HDL ratio gradually increased with increasing serum ALP quartile in both men and women (*p*-value < 0.001).

The ORs (95% CIs) for high TG/HDL ratio according to serum ALP quartile are shown in Table 2. Compared with the lowest quartile, the OR (95% CI) of the highest ALP quartile for high TG/HDL was 1.517 (1.234–1.866) in men and 1.881 (1.399–2.528) in women after adjusting for age, sex, BMI, SBP, DBP, AST, ALT, fasting glucose, smoking status, alcohol consumption, resistance exercise, household income, and educational level (post hoc *p*-value < 0.001).

### 3.3. Association between TyG Index and ALP

**Figure 3:**
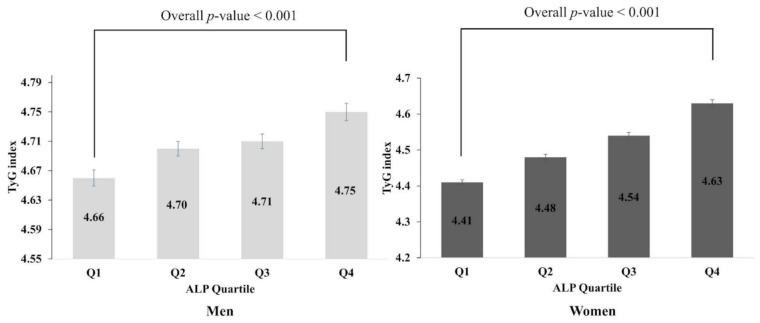
Trend analysis of TyG index according to ALP quartile. Values are assessed by ANOVA.

Figure 3 illustrates the analysis trend for TyG index according to ALP quartile in men and women. The mean TyG index increased significantly according to serum ALP quartile in both sexes (*p* value < 0.001).

The ORs (95% CIs) for high TyG index according to serum ALP quartile are presented in Table 3. Compared with the lowest quartile, the OR (95% CI) of the highest ALP quartile for high TyG index was 1.374 (1.093–1.728) in men and 2.047 (1.468–2.855) in women after adjusting for age, sex, BMI, SBP, DBP, AST, ALT, fasting glucose, smoking status, alcohol consumption, resistance exercise, household income, educational level, and menopause status (post hoc *p*-value < 0.001).

## 4. Discussion

In this nationally representative cross-sectional study, we investigated the association of serum ALP with insulin resistance in community-dwelling Korean adults. We used TG/HDL-C ratio and TyG index to assess insulin resistance. Emerging evidence has identified the TG/HDL-C ratio and TyG index as markers for insulin resistance with high sensitivity and specificity [6,7,8]. In the present study, we showed that serum ALP is positively associated with the TG/HDL-C ratio and TyG index even after adjusting for potential confounding factors of age, sex, BMI, SBP, DBP, hepatic enzymes, smoking status, alcohol consumption, resistance exercise, household income, and educational level. In particular, women showed a stronger association between serum ALP and insulin resistance surrogates than men. Additionally, HOMA-IR significantly increased according to ALP quartiles in both men and women.

Previous studies have linked serum ALP and insulin resistance, although the association is still controversial [20,21,22]. Gurler et al. suggested that there was no significant difference between ALP levels in 124 female patients with or without insulin resistance [23]. Additionally, several studies reported that there was no significant association between ALP and incident diabetes [20,24,25]. However, our results are consistent with previous findings by Kim et al. who showed a significant association between increased ALP levels and metabolic syndrome [15]. Moreover, among components of metabolic syndrome, hypertriglyceridemia and low HDL cholesterol were found to be significantly increased according to ALP levels in 14,224 Korean subjects [22]. Similarly, positive association for insulin and glucose metabolism with ALP levels were also reported in children and adolescents [26]. Although the reason for inconsistent results regarding the association between ALP and insulin resistance is unclear, sex, ethnicity, and study population size may lead to the discrepancies. Our study and a previous study consistently showed a stronger relationship between ALP levels and insulin resistance or metabolic syndrome in women compared to men [15].

ALPs are ubiquitous ectoenzymes, which hydrolyze monophosphate ester. ALPs have many substrates and participate in various metabolic and biosynthetic pathways [27]. Gene knockout studies have helped define some of functions of each isoenzyme in bone, teeth, the central nervous system, and in the gut [27]. For example, mice and humans with inactivated ALPL gene mimic a severe form of hypophosphatasia [28]. Hypophosphatasia is a rare disorder induced by a mutation in the ALPL gene resulting in a diminished activity of the enzyme in target tissues. Young infants with hypophosphatasia present various symptoms ranging from impaired mineralization at birth, bone pain, leg bowing, recurrent fractures, and muscular insufficiency, to tooth loss in adults [29]. In hypophosphatasia, three phosphocompounds, including phosphoethanolamine (PEA), inorganic pyrophosphate (PPi), and pyridoxal 5’-phosphate (PLP), accumulated endogeneously and appear to be natural substrates of TNSALP [30,31]. Although PEA remains controversial as an endogenous substrate of TNSALP, it is commonly used in the clinics as a diagnostic marker for hypophosphatasia. Among these substrates, PPi plays a major role in preventing calcification; therefore, elevated PPi levels induce skeletal diseases. In order to restore the level of PPi, ectonucleotide pyrophosphatase/phosphodiesterase 1 (ENPP1) and progressive ankyloses protein are involved in the physiological mineralization [32,33,34]. These proteins and ALP regulate and finetune the formation of hydroxyapatite crystals, maintaining the PPi/Pi ratio for normal bone mineralization [35]. Lack of ENPP1 can cause severe depletion of PPi and arterial calcification in infancy [36]. Additionally, Lomashvile et al. showed that upregulated TNSALP in rats leads to the hydrolysis and, therefore, inactivation of PPi, which is a potent inhibitor of hydroxyapatitie crystal growth and a potential inhibitor of vascular calcification [37].

Elevation of ALP levels are generally associated with cholestatic liver disease, such as biliary obstruction due to cancer, choledocholithiasis, biliary stricture, sclerosing cholangitis, drug-induced liver injury, and hepatitis. Additionally, bone ALP, which is a marker of bone formation, is not only involved in physiological but also in pathological mineralization, including Paget’s disease, osteogenic sarcoma, healing fracture, osteomalacia, hyperparathyroidism, hyperthyroidism, chronic kidney disease, and vascular calcification [38]. Since various diseases are characterized by high ALP activity, measuring ALP alone without assessment of other metabolic parameters is insufficient as a surrogate for insulin resistance. Nonetheless, the results of this study may have clinical implication because, if the clinician has confirmed that there is no liver or bone-related diseases, insulin resistance or metabolic syndrome can be considered by seeing elevated ALP levels.

Chronic subclinical inflammation plays an important role in the link between ALP and metabolic syndrome. Seo et al. reported that inflammatory markers such as C-reactive protein (CRP) and leukocyte count increased in accordance with serum ALP quartile in older adults [18]. Additionally, several previous studies have shown that serum ALP level is positively associated with CRP concentration [39,40,41]. However, little is known about the relationship between serum ALP and insulin resistance, another crucial pathophysiology of metabolic syndrome. Our findings support the idea that insulin resistance contributes to the association between serum ALP and metabolic syndrome as well as chronic low-grade inflammation.

The underlying mechanisms for the association between insulin resistance and ALP remain uncertain, and several possible mechanisms are under consideration. A previous study reported that serum ALP level is higher in obese people [42]. East Asians generally have higher body fat percentages than non-Asians at the same BMI. In this regard, our study population shows a relatively low BMI with high ALP levels. ALP activity has been shown to be involved in adipogenesis in both experimental and human studies [43,44]. Hernández-Mosqueira et al. found that the gene encoding TNSALP is expressed in adipose tissue and adipocytes. They found that serum ALP level was inversely associated with the level of adiponectin, which is closely related to development of type 2 diabetes and hypertension [44]. Additionally, knocking down the ALPL gene decreased the expression of leptin, which plays a key role in adipocyte systemic signaling and insulin resistance [45]. These results suggest that an increased TNSALP level contributes to insulin resistance by increasing leptin level and reducing adiponectin level in a direct or indirect manner. Furthermore, high ALP activity promotes lipolysis, which is associated with insulin resistance by increased free fatty acids levels [46]. Cheung et al. reported that serum insulin level was positively associated with bone-specific ALP based on HOMA-IR for pancreatic cell function in 3773 nondiabetic participants [47]. Another possible mechanism is link between elevated ALP and low vitamin D. High level of ALP has been reported to reflect decreased levels of vitamin D in several studies [48,49]. Low vitamin D levels are associated with smooth muscle cell proliferation, endothelial dysfunction, vascular inflammation, vascular calcification, and atherosclerosis [50,51].

Conversely, intestinal ALP was reported to prevent induction of metabolic syndrome by inhibiting uptake of endotoxin (lipopolysaccharides) in high-fat diet-fed mice [52]. However, intestinal ALP contributes to only ~10% of total ALP activity in healthy individuals, thus the results of our findings would not be mainly affected by intestinal ALP activity. Further studies are required to demonstrate the association between each ALP isozyme and metabolic syndrome.

This study has several limitations. First, insulin resistance was assessed by surrogate markers of TyG index and TG/HDL-C ratio instead of the gold standard, hyperinsulinemic-euglycemic clamp. However, accumulating evidence has validated the sensitivity and specificity of these indirect markers [6,7]. Previous cross-sectional study also showed HOMA-IR, which is a representative marker of insulin resistance, increased according to ALP quartiles in both men and women [15]. Second, since the data were observational and collected using a cross-sectional design, it is difficult to conclude causation. Although a large prospective study showed association between ALP and incident type 2 diabetes, there are a limited number of studies showing a direct link between ALP and insulin resistance. Therefore, further prospective and experimental studies are required to verify the direct mechanistic association between insulin resistance and ALP. Third, we measured total ALP activity for the quantitation according to the recommendation of the International Federation of Clinical Chemistry and Laboratory Medicine. These ALP enzymatic assays cannot discriminate the ALP isozymes and isoforms, thus we could not conduct subgroup analysis by each ALP isozyme. Enzyme measurement is also defined as “catalytic amount”, which is the amount of an agreed-upon substrate that is converted to the product in an agreed-upon measurement system [53]. The measurement process depends on the experimental conditions, such as temperature, pH, and the nature of the buffer [54]. Therefore, serum ALP activity does not reflect the actual ALP concentration. Fourth, this data does not contain information about serum bilirubin or abdomen ultrasound result, so we could not exclude the possibility of biliary diseases such as gallbladder stone, cholangitis, or cholecystitis. To minimize this limitation, we adjusted for the hepatobiliary markers such as AST and ALT as confounding factors. Despite these potential limitations, our results offer potential clinical implications. This is the first nationwide, general population study to show a positive association between serum ALP level and TG/HDL ratio and TyG index after adjusting for confounding factors. Two previous studies about metabolic syndrome and ALP showed that low HDL-C and hypertriglyceridemia among components of metabolic syndrome were significantly associated with ALP [15,22]. Our results are consistent with these studies. Additionally, compared to TG/HDL ratio or TyG index, ALP is an economical marker and can be collected regardless of whether the patient was fasting or not in routine clinical practice. Therefore, our results may have clinical implications because clinicians may consider insulin resistance or metabolic syndrome by detecting an elevated ALP levels.

## 5. Conclusions

In conclusion, serum ALP activity was positively and independently correlated with TyG index and TG/HDL-C ratio. Since serum ALP level can be affected by various factors, such as patient’s age, renal, musculoskeletal, and hepatobiliary diseases, ALP cannot be used alone as an insulin resistance surrogate. Therefore, serum ALP can be used as a complementary measure in the evaluation of insulin resistance or metabolic syndrome alongside with TyG index or TG/HDL ratio.

## Figures and Tables

**Table 1:** Clinical characteristics of the study population.

	Men ALP Quartile (U/L)					Women ALP Quartile (U/L)				
	Q1 (55–190)	Q2 (191–224)	Q3 (225–265)	Q4 (266–923)	*p*-Value	Q1 (48–161)	Q2 (162–198)	Q3 (199–245)	Q4 (246–790)	*p*-Value
*n*	1489	1482	1496	1472	5939	1722	1732	1744	1731	6929
Age (years)	42.2 ± 0.4	42.5 ± 0.4	42.4 ± 0.5	42.9 ± 0.5	0.702	37.4 ± 0.3	39.0 ± 0.4	43.1 ± 0.5	50.4 ± 0.5	<0.001
BMI (kg/m2)	24.0 ± 0.1	24.2 ± 0.1	24.0 ± 0.1	24.2 ± 0.1	0.445	22.1 ± 0.1	22.8 ± 0.1	23.4 ± 0.1	24.3 ± 0.1	<0.001
SBP (mmHg)	120.1 ± 0.5	120.6 ± 0.5	121.4 ± 0.5	122.8 ± 0.5	<0.001	108.6 ± 0.4	111.5 ± 0.4	115.3 ± 0.5	121.7 ± 0.5	<0.001
DBP (mmHg)	79.9 ± 0.3	80.4 ± 0.4	80.5 ± 0.3	81.3 ± 0.4	0.032	71.5 ± 0.3	73.0 ± 0.3	74.4 ± 0.3	76.7 ± 0.3	<0.001
Fasting plasma glucose (mg/dL) (70–99 mg/dL)	96.2 ± 0.5	97.6 ± 0.7	97.1 ± 0.6	101.4 ± 1.0	<0.001	89.8 ± 0.3	91.7 ± 0.4	93.3 ± 0.4	99.1 ± 0.7	<0.001
Insulin (μIU/mL) (2.6–24.9 μIU/mL)	9.8 ± 0.2	10.1 ± 0.2	10.5 ± 0.3	11.1 ± 0.3	0.001	9.3 ± 0.1	10.1 ± 0.2	10.3 ± 0.2	11.1 ± 0.3	<0.001
HOMA-IR	2.4 ± 0.0	2.5 ± 0.1	2.6 ± 0.1	2.8 ± 0.1	0.001	2.1 ± 0.0	2.3 ± 0.0	2.4 ± 0.1	2.9 ± 0.1	<0.001
Triglycerides (mg/dL) (<150 mg/dL)	142.0 ± 3.9	155.5 ± 4.5	160.6 ± 4.0	170.9 ± 5.2	<0.001	86.1 ± 1.3	99.8 ± 1.8	111.6 ± 2.3	131.3 ± 2.6	<0.001
HDL-C (mg/dL) (>60 mg/dL)	48.0 ± 0.3	46.3 ± 0.3	45.8 ± 0.3	45.2 ± 0.3	<0.001	53.9 ± 0.4	52.8 ± 0.3	52.0 ± 0.3	49.8 ± 0.4	<0.001
ALT (U/L) (≤35 U/L)	23.3 ± 0.4	25.4 ± 0.5	26.6 ± 0.6	28.6 ± 0.6	<0.001	13.6 ± 0.2	15.0 ± 0.2	17.1 ± 0.3	20.6 ± 0.4	<0.001
AST (U/L) (≤40 U/L)	21.9 ± 0.2	23.0 ± 0.3	23.7 ± 0.3	25.1 ± 0.3	<0.001	17.2 ± 0.1	18.1 ± 0.1	19.5 ± 0.2	22.1 ± 0.3	<0.001
WBC μL (4000–10000/μL)	6065.5 ± 41.5	6315.2 ± 40.9	6384.0 ± 44.7	6464.6 ± 44.1	<0.001	5455.4 ± 39.4	5636.7 ± 39.8	5777.3 ± 44.1	5866.1 ± 40.6	<0.001
Current smoking (%)	535 (46.2)	561 (45.7)	581 (47.5)	591 (50.1)	0.654	100 (6.7)	76 (5.7)	67 (5.0)	71 (4.6)	0.106
Alcohol drinking (%)	646 (41.7)	572 (37.3)	552 (5.1)	488 (32.5)	<0.001	216 (12.7)	199 (12.1)	143 (9.2)	96 (6.4)	<0.001
Resistance exercise (%)	370 (24.0)	294 (19.3)	320 (22.0)	296 (20.2)	0.046	176 (9.6)	136 (7.7)	151 (8.1)	121 (6.4)	0.023
Household income (%)					<0.001					<0.001
Quartile 1 (Lowest)	190 (10.7)	187 (9.6)	241 (13.4)	315 (16.5)		161 (9.3)	234 (13.0)	315 (16.2)	443 (23.4)	
Quartile 2	345 (24.5)	361 (25.0)	369 (26.8)	367 (25.4)		412 (25.7)	409 (25.3)	422 (25.7)	464 (27.4)	
Quartile 3	424 (29.2)	453 (32.9)	443 (30.6)	407 (30.7)		577 (32.1)	516 (30.1)	512 (31.0)	454 (28.3)	
Quartile 4	512 (35.6)	465 (32.5)	428 (29.2)	365 (26.2)		600 (32.8)	570 (31.6)	470 (27.2)	373 (20.9)	
Education level (%)					<0.001					<0.001
Elementary school	177 (8.1)	202 (9.7)	223 (10.8)	288 (13.0)		118 (6.0)	246 (11.1)	438 (20.0)	757 (38.2)	
Middle school	149 (9.9)	159 (8.6)	169 (10.8)	223 (13.0)		120 (7.3)	157 (8.6)	195 (11.4)	210 (13.0)	
High school	553 (40.2)	566 (43.2)	529 (41.0)	529 (43.9)		749 (42.5)	725 (44.3)	626 (39.8)	463 (29.9)	
≥College	591 (41.8)	532 (38.5)	546 (37.4)	400 (30.2)		757 (44.1)	604 (36.1)	455 (28.8)	286 (18.8)	
Hypertension (%)	293 (14.5)	275 (12.9)	296 (14.2)	311 (15.4)	0.594	92 (4.1)	172 (7.7)	304 (14.1)	491 (24.5)	<0.001
Impaired fasting glucose (%)	398 (23.4)	360 (20.9)	351 (20.9)	364 (22.2)	0.408	172 (9.5)	219 (11.5)	306 (15.1)	389 (21.0)	<0.001
Diabetes Mellitus (%)	113 (5.6)	115 (6.2)	97 (4.2)	131 (6.7)	<0.001	43 (1.9)	74 (3.2)	97 (4.2)	159 (8.0)	<0.001
Obese (%)	170 (20.3)	186 (21.0)	169 (22.0)	194 (24.8)	0.293	86 (15.0)	166 (24.3)	213 (27.4)	330 (35.1)	<0.001
Metabolic syndrome (%)	281 (16.6)	287 (16.5)	317 (18.9)	374 (23.9)	<0.001	119 (5.6)	245 (12.1)	358 (17.0)	599 (32.0)	<0.001

Abbreviation: BMI, body mass index; SBP, systolic blood pressure; DBP, diastolic blood pressure; HDL-C, high-density lipoprotein-cholesterol; AST, aspartate aminotransferase; ALT, alanine aminotransferase; WBC, white blood cell count. Values are presented as mean ± standard deviation or number (percentage). *p*-values were assessed by weighted analysis of variance or weighted chi-square test.

**Table 2:** Odds ratios and 95% confidence intervals for high TG/HDL ratio according to serum ALP.

	ALP Quartile (U/L)				
	Q1	Q2	Q3	Q4	Overall *p*-Value
Male					
Model 1	Reference	1.250 (1.039–1.503)	1.518 (1.246–1.850)	1.673 (1.384–2.024)	<0.001
Model 2	Reference	1.196 (0.990–1.444)	1.425 (1.159–1.750)	1.473 (1.204–1.802)	<0.001
Model 3	Reference	1.214 (1.003–1.470)	1.473 (1.195–1.816)	1.517 (1.234–1.866)	<0.001
Female					
Model 1	Reference	1.483 (1.115–1.972)	1.602 (1.228–2.089)	2.272 (1.720–3.002)	<0.001
Model 2	Reference	1.431 (1.073–1.909)	1.517 (1.156–1.991)	1.970 (1.479–2.622)	<0.001
Model 3	Reference	1.419 (1.064–1.894)	1.493 (1.133–1.967)	1.881 (1.399–2.528)	<0.001

Model 1: age, BMI; Model 2: age, BMI, SBP, DBP, AST, ALT, fasting glucose; Model 3: age, BMI, SBP, DBP, AST, ALT, fasting glucose, smoking status, alcohol consumption, resistance exercise, household income, and educational level.

**Table 3:** Odds ratios and 95% confidence intervals for high TyG index according to serum ALP quartile.

	ALP Quartile (U/L)				
	Q1	Q2	Q3	Q4	Overall *p*-Value
Male					
Model 1	Reference	1.151 (0.948–1.396)	1.419 (1.169–1.722)	1.584 (1.290–1.946)	<0.001
Model 2	Reference	1.080 (0.873–1.336)	1.353 (1.099–1.665)	1.293 (1.033–1.619)	<0.001
Model 3	Reference	1.109 (0.893–1.376)	1.448 (1.168–1.794)	1.374 (1.093–1.728)	<0.001
Female					
Model 1	Reference	1.658 (1.243–2.210)	1.787 (1.332–2.396)	2.597 (1.953–3.454)	<0.001
Model 2	Reference	1.534 (1.127–2.088)	1.667 (1.211–2.296)	2.017 (1.456–2.793)	<0.001
Model 3	Reference	1.590 (1.164–2.171)	1.767 (1.284–2.432)	2.047 (1.468–2.855)	<0.001

Model 1: age, BMI; Model 2: age, BMI, SBP, DBP, AST, ALT, fasting glucose; Model 3: age, BMI, SBP, DBP, AST, ALT, fasting glucose, smoking status, alcohol consumption, resistance exercise, household income, and educational level.

## Data Availability

The KNHANES data are publicly available through the KNHANES website (https://knhanes.kdca.go.kr/knhanes (accessed on 5 October 2020)).
